# Cancer During Pregnancy: The Role of Vascular Toxicity in Chemotherapy-Induced Placental Toxicity

**DOI:** 10.3390/cancers12051277

**Published:** 2020-05-18

**Authors:** Hadas Bar-Joseph, Fedro Alessandro Peccatori, Tal Goshen-Lago, Fulvia Milena Cribiù, Giovanna Scarfone, Irit Miller, Luba Nemerovsky, Mattan Levi, Ruth Shalgi, Irit Ben-Aharon

**Affiliations:** 1 The Transgenic Mice, Cryopreservation and Assisted Reproduction (TMCR) Unit, Veterinary Service Center, Tel Aviv University, Ramat Aviv, 69978 Tel Aviv, Israel; hadasbj@gmail.com; 2 Gynecologic Oncology Program, European Institute of Oncology IRCCS, 20122 Milan, Italy; fedro.peccatori@ieo.it; 3 Oncology, Rambam Health Care Center, 3109601 Haifa, Israel; T_GOSHENLAGO@rambam.health.gov.il; 4 Pathology Unit, Fondazione IRCCS Ca’ Granda Ospedale Maggiore Policlinico, 20122 Milan, Italy; fulviamilena.cribiu@policlinico.mi.it; 5 Department of Gynecological Oncology, Fondazione Cà Granda Policlinico Ospedale Maggiore of Milano, 20122 Milan, Italy; giovi.scarfone@gmail.com; 6 Department of Cell and Developmental Biology, Sackler Faculty of Medicine, Tel-Aviv University, Ramat-Aviv, 69978 Tel-Aviv, Israel; iritmil@gmail.com (I.M.); lnemerovsky@gmail.com (L.N.); mattanlevi@gmail.com (M.L.); shalgir@tauex.tau.ac.il (R.S.); 7 Technion Integrated Cancer Center (TICC), Faculty of Medicine, Technion, 3200003 Haifa, Israel

**Keywords:** doxorubicin, embryo, pregnancy, placenta, vascular toxicity

## Abstract

Breast cancer is diagnosed in ~0.3% of pregnant women. Studies that have addressed gestational and neonatal outcomes of chemotherapy during pregnancy have demonstrated increased gestational complications including preeclampsia and intrauterine growth retardation. We hypothesized that anthracycline-induced gestational complications could be derived from direct toxicity on the placenta vasculature. Pregnant ICR mice (day E12.5) were treated with doxorubicin (DXR; 8 mg/kg) or saline, while their umbilical cord blood flow was imaged by pulse-wave (PW) Doppler. Mice were euthanized on day E18.5, and their embryos and placentae were collected for further analysis. Unlike control mice, the DXR-treated mice presented an acute change in the umbilical cord’s blood flow parameters (velocity time integral and heart rate interval), reduced embryos’ weight, reduced placenta efficiency, and modulation in vascular-related pathways of treated placenta proteomics. Apoptosis and proliferation were also enhanced, as demonstrated by TUNEL and proliferating cell nuclear antigen (PCNA) analysis. We further examined the placentae of patients treated with epirubicin (EPI), who had been diagnosed with breast cancer during pregnancy (weeks 27–35). The immunohistochemistry of the EPI-treated human placentae showed enhanced proliferation and apoptosis as compared with matched chemo-naïve placentae, as well as reduced neovascularization (CD34). Our findings suggest that anthracycline-induced vascular insult promotes placental toxicity, and could point to potential agents designated to offset the damage and to reduce gestational complications in pregnant cancer patients.

## 1. Introduction

Breast cancer (BC) is the most commonly diagnosed malignancy during pregnancy with an estimated incidence of one in 3000 pregnancies [1,2]. The incidence of cancer during pregnancy is expected to rise further in developed countries, due to an increase in the mean maternal age at pregnancy and to an increasing incidence of cancer in young patients throughout the last decade [3,4].

BC diagnosed during pregnancy represents a unique clinical scenario, which requires a delicate balance between risks and benefits for the mother and fetus [5].

While surgery can be performed at any gestational age without embryo-fetal impairment, endocrine treatment, anti-HER2 (Human Epidermal Growth Factor Receptor 2) therapy, and breast irradiation are usually postponed until after delivery [6].

Data about the obstetrical and fetal safety of chemotherapy administered during pregnancy are still not conclusive [7,8]. Several studies have reported higher rates of neonatal complications after in utero exposure to anticancer drugs [1,2,9], whereas others have found no significant differences [10,11]. The unwanted effects of chemotherapy administered during pregnancy depend mainly on the gestational stage; exposure during the first trimester is associated with a 10–20% risk of fetal malformations, whereas exposure during the second and third trimesters is significantly safer, with a fetal malformation rate similar to the unexposed population [12]. Some of the effects on newborns can be referred, at least partially, to iatrogenic prematurity, i.e., the induction of anticipated delivery for medical reasons [1,2]. Others can be directly related to the chemotherapy administered and include premature labor, premature rupture of membranes (PROM), and intrauterine growth restriction (IUGR) [12,13,14]. However, other factors such as mothers’ stress or unsuitable nutrition during the pregnancy could also contribute.

The most frequent drugs administered for BC during pregnancy are anthracyclines (mainly doxorubicin; DXR or epirubicin; EPI) [15] and taxanes, which have been measured in low concentrations in fetal plasma. DXR has an acceptable safety profile; it has been hardly detected in the amniotic fluid [16], and it has been documented to induce only a few short-term complications [17].

We previously demonstrated, in non-pregnant mice and women, that DXR elicits direct acute endothelial damage that initiates vascular toxicity, which can progress to long-term chemotherapy-induced vascular morbidity. Using real-time in vivo mice imaging, we characterized patterns of chemotherapy-induced vascular toxicity, manifested by impaired blood flow [18], and demonstrated that anticoagulants reduced the observed vascular impairment [19]. Acute vasculature impairment of the gonads, examined in a preclinical setting in both female and male mice, induced gonadal toxicity [18,19]. We further studied this phenomenon in a clinical setting, where we followed ovarian blood flow in a cohort of young BC patients treated with DXR-based regimens, and showed that DXR induced an age-dependent ovarian toxicity, which resembled our findings in the animal model [20]

In the current study, we examined the hypothesis that anthracyclines could induce placental vascular toxicity which can lead to impaired placenta function and could determine gestational and fetal complications. We administered DXR to pregnant mice during a gestational age corresponding to a human’s second trimester (day E12.5) and explored its immediate effect on the umbilical cord blood flow and the later effects on the placenta and embryo at full term. Moreover, we analyzed placenta histology and immunohistochemistry in a sample of women who received EPI-based chemotherapy during pregnancy.

## 2. Results

### 2.1. Blood Flow in the Umbilical Cord

**Figure 1:**
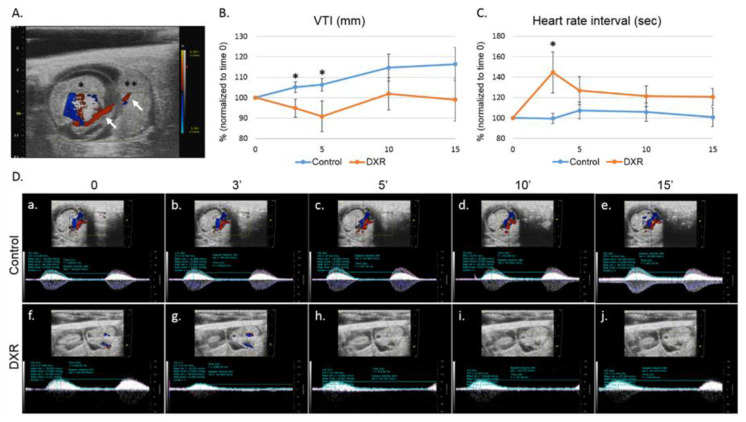
Blood flow in the umbilical cord. (**A**) On day E12.5 of pregnancy, an umbilical cord artery of one embryo was viewed by Color Doppler mode; presentation of an embryo (*) with its adjacent placenta (**) and the umbilical cord (arrows) in which the acquisition was taken. Following a short stabilization period, a baseline arterial blood flow was recorded and quantified. Then, mice were injected with either doxorubicin (DXR; 8 mg/kg, *n* = 7, *n* = number of imaged arteries, one embryo per pregnant mouse) or saline (control; *n* = 6). The arterial blood flow was monitored continuously by pulse-wave (PW) Doppler mode for 15 min, recorded and analyzed at various time points post injection by analyzing the (**B**) velocity time integral (VTI); and (**C**) heart rate interval. (**D**) Representative PW Doppler photos of the analyzed time points. Analysis of each PW Doppler cine loop was performed. Values of post-treatment imaging were normalized according to pretreatment imaging values of each mouse (defined as 100%). Saline-injected mice were standardized to 100% as a reference to DXR-injected mice. Data are expressed as mean ± standard error of mean (SEM). Individual comparisons were made using a Student’s t-test. (*) *P* value of 0.05 was considered statistically significant.

We used pulse-wave (PW) Doppler to image the blood flow in the umbilical cord of pregnant mice (on day E12.5 of pregnancy, Figure 1A) and followed the changes in the velocity time integral (VTI, mm) and in the heart rate interval, before and after injecting DXR or saline (control). Five minutes after DXR administration, the VTI value measured in the DXR-injected mice was reduced to 90% of its pretreatment value, whereas the VTI value in the control mice increased by 5%, though not in a statistically significant way. The same trend was observed 15 minutes after drug administration (Figure 1B,D). We also found that the heart rate interval in the DXR-injected mice increased by 40% over the pretreatment values, 3 minutes post administration and no change was observed in the control mice (Figure 1C,D).

### 2.2. Embryos and Placentae Weights

**Figure 2:**
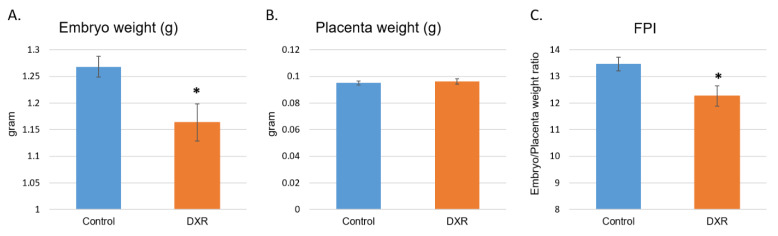
Embryos and placentae weight. On day E18.5, 6 days after injection of either DXR or saline, embryos and placentae were removed from the uterus (*n* = 52, from 5 DXR-treated pregnant mice and *n* = 61, from 5 control pregnant mice). Each embryo (**A**) and its placenta (**B**) were weighed separately (gram); fetal to placenta weight ratio index (FPI) was calculated (**C**). Data are expressed as mean ± SEM. Control and DXR groups presented normal distribution of their litter size, embryos’ and placentae’ weights, and the FPI values, by the Kolmogorov–Smirnov test. The litter size of the two groups is not statistically different from one another or from the average litter size of ICR (Institute of Cancer Research) females (reported by Envigo). Individual comparisons were made using a Student’s t-test. (*) *P* value of 0.05 was considered statistically significant.

The entire embryonic sacs were removed from the DXR- and saline-treated pregnant mice on day E18.5, embryos were separated from the placentae and both were weighed (Table S1). A significant decrease in the weight of the DXR-exposed embryos was observed as compared with that of the control embryos (Figure 2A, mean weight of 1.163 g and 1.268 g, respectively, 8.2% difference). Unlike the embryos, the weights of the placentae in both treatment groups were the same (Figure 2B). The fetal to placenta weight ratio index (FPI), which represents placental efficiency [21], was significantly reduced in the DXR-injected mice as well (Figure 2B, 8.9% difference).

### 2.3. Proteomic Analysis of the Placentae

**Figure 3:**
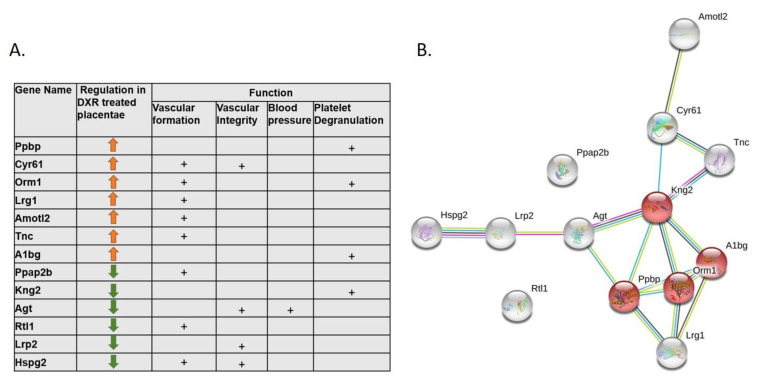
Proteomic analysis of DXR or saline-treated placentae (control samples). Proteins from four DXR- or saline-exposed placentae (from 3 pregnant mice in each group) were identified, quantified, and their intensities were normalized. (**A**) Summary table of proteins that exhibit significant changes in their intensity (false discovery rate (FDR) < 0.05) and are potentially related to vascular toxicity; (**B**) STRING (Search Tool for Retrieval of Interacting Genes/Proteins) database analysis, as elaborated in the Methods section, of functional associations network of the vascular toxicity related proteins. Platelet degranulation pathway proteins are colored in red.

Proteomic analysis of mice placentae revealed a significant change in the expression of 277 proteins upon exposure to DXR (Table S2). Thirteen of them are involved with vascular changes and the related placental toxicity; some of them participate in the platelet degranulation pathway, thereby contributing to increased procoagulant activity (Kng2, A1bg, Ppbp, Orm1; Reactome pathway MMU-114608) or to regulation of neo-vascular formation, vascular-wall integrity, and blood pressure (Cyr61, Lrg1, Amotl2, Tnc, Ppap2b, Agt, Lrp2, Rtl1, and Hspg2) (Figure 3A,B).

### 2.4. Histological Evaluation of Placentae

**Figure 4:**
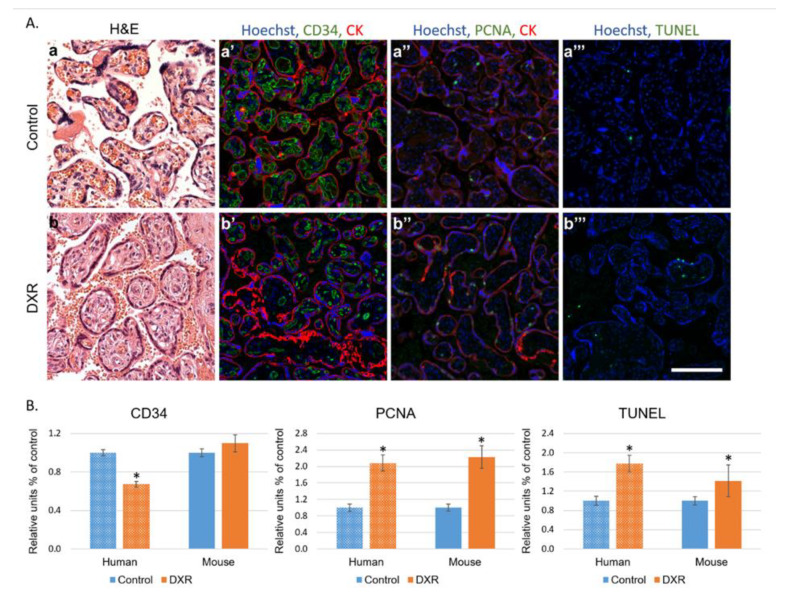
Histology of placentae. (**A**) Human placentae from women diagnosed with breast cancer during pregnancy (week 27–35) and treated with EPI (*n* = 7) were compared with human placentae from chemo-naïve women (control *n* = 10). Representative pictures of control- (a–a‴) or EPI-exposed (b–b‴) human placenta, stained for the following: H&E (a,b); CD34 (neovascularization a′,b′); PCNA (proliferating cell nuclear antigen; a″,b″); pan-cytokeratin (positive control for staining; CK a′,a″,b′,b″); or TUNEL (apoptosis a‴,b‴). Florescence images were photographed using a LSM-510 confocal laser-scanning microscope. Offset calibration of the photomultiplier was performed with sections stained with secondary antibodies only. Bar = 100 µm. (**B**) Florescence pattern was imaged in human and in dissections of placentae from pregnant female mice treated with either DXR (*n* = 52, from 4 pregnant mice) or saline (*n* = 21, from 4 pregnant mice). The average number of PCNA positive cells, TUNEL positive cells, or CD34 blood vessels were automatically analyzed by FIJI software, and compared with control samples (defined as 1). Data are expressed as mean ± SEM. Individual comparisons were made using a Student’s t-test. (*) *P* value of 0.05 was considered statistically significant.

Histological sections of human (Table S3) and mouse placentae were evaluated for neovascularization, proliferation, and apoptosis (CD34 and proliferating cell nuclear antigen (PCNA) immunostaining and TUNEL (terminal deoxynucleotidyl transferase dUTP nick end labeling) staining, respectively, Figure 4A). In humans, the analysis depicted a different pattern of vascular landscape in the placentae of EPI-treated patients, manifested by decreased neovascularization and enhanced proliferation and apoptosis as compared with gestational age-matched placentae of chemo-naïve untreated women (Figure 4A,B). In mice, the changes resembled those observed in humans; both proliferation and apoptosis rates were higher in placentae of the DXR-exposed mice than in those of the control mice. CD34 staining indicated no difference in the number of newly formed blood vessels between placentae of both populations (Figure 4B and Figure S1).

## 3. Discussion

Our findings suggest that anthracycline-induced gestational complications are derived in part, from a direct and acute impairment of the placenta’s vasculature, along with the possible direct effect on the placenta cellular compartments and the embryo itself. We characterized the vascular-derived placental toxicity in preclinical and clinical settings using real-time intravital imaging, immunofluorescence profiling, and transcriptome analysis of the placentae. These findings are in agreement with our previous work, performed in a mice model and in young BC patients, presenting a direct and immediate effect of DXR on the peripheral vasculature [20]. Moreover, DXR-induced impairment of the gonads’ vasculature and blood supply acts as an underlying mechanism preceding gonadal toxicity [18,19].

The largest European study, which followed obstetrical outcomes of 413 patients diagnosed with BC during pregnancy, among whom 197 patients were treated during pregnancy (76 with anthracyclines) [15], shows that infants exposed in utero to chemotherapy had a lower birthweight and were more inclined towards IUGR and PROM incidents. To note, these differences were attributed by the authors to prematurity, as half of the cohort delivered preterm, with 23% before the 35th week of gestation. Other studies have also demonstrated an increased rate of gestational complications. However, data regarding late side effects are still limited.

A prospective study that followed 96 children, who had been exposed in utero to chemotherapy, and evaluated their general health status, growth, cognitive development, cardiac structure, and function at early childhood (12–42 month), concluded that prenatal exposure did not significantly impair pediatric development [11]. However, the follow-up period was too short to determine long-term cardiotoxicity and neurocognitive deficits, which could become more apparent later in life. Additionally, a study that followed 70 children until the age of 18, reported that cognitive development scores were lower for children who were born preterm than for those born at full term, independent of cancer treatment. Moreover, due to subtle changes in cardiac measurements they emphasized the need for longer follow-up [10].

Anthracyclines serve as a cornerstone chemotherapy in the treatment of breast cancer during pregnancy [17,22]. DXR and EPI exert anti-neoplastic activity via several mechanisms such as: they inflict damage to the DNA of proliferating cells, injure endothelial cells [23], and elevate reactive oxygen species (ROS) [24]. Two known anthracycline-related phenomena are vascular toxicity and long-term cardiomyopathy [18,23,24].

The placenta is a unique organ that serves as a filter of substances in which drug transport can change during gestation and is tightly regulated [16]. Early reports on DXR transfer via the placenta have indicated that DXR and its metabolites were found in fetal tissues [22], however, administration of DXR on the second and third trimesters was considered to have a good safety profile for the embryo [16,22]. When low doses of DXR were administered, the drug levels were undetectable in amniotic fluid, fetal brain, gastrointestinal tract, or in cord blood retrieved from a healthy child born 48 hours after drug administration [16]. Thus, reinforcing our hypothesis that the observed gestational complications are derived, in part, from the impairment of the placenta’s vasculature and function.

Former studies have indicated that several frequent gestational complications (such as preeclampsia and IUGR) observed in chemotherapy-treated patients, were derived from placental vascular alterations. In our study, we explored the effect of DXR administration, during mice pregnancy at a gestational age that corresponds to the second trimester of human pregnancy on the umbilical cord blood flow, and its influence on the placenta and the embryo. Intravenous (IV) injection of DXR resulted in an immediate but transient decrease in VTI, and an increase in heart rate interval (relative bradycardia). VTI indicates the distance that the blood travels during a certain cardiac cycle; the physiologic response to IV administration of fluids is a consistent increase in VTI [25]. A decrease in VTI, as occurred immediately after DXR administration, along with increased heart rate interval, can indicate an altered umbilical cord blood flow. These data correlate with our former reports regarding DXR-induced gonadal and vascular toxicity [18,26], which implied that the same pattern of anthracycline-induced vascular impairment applies also in the placenta. This acute effect instigated a cascade of events that were initially translated into reduced blood flow, followed by a lower birth weight of mice embryos exposed to DXR in utero as compared with control embryos.

Several studies have highlighted an increased frequency of lower birth weight following chemotherapy during pregnancy, and a higher tendency to preterm deliveries [2]. However, as mentioned above, the correlation between the newborn’s weight and exposure to chemotherapy is not linear. In the present study, all embryos were delivered on the same gestational day (E18.5, 6 days after saline or DXR administration), ruling out these parameters. Thus, we can conclude that the observed weight difference is a result of the chemotherapy administered to the pregnant mouse. Placentae weights did not differ between the two groups; nonetheless, the significant reduction of the FPI suggests a compromised placental function.

Upon disruption of the vasculature, hemostasis is activated by vasoconstriction, platelet plug formation, and coagulation [27], and followed by formation of new vasculature. Proteomic analysis of the placentae, following DXR treatment, revealed several cellular pathways that were modified and could point to a vascular damage. Among the altered proteins, we detected the following: A1bg which participates in the initial platelet adhesion to the vascular subendothelium after vascular injury, Orm1 which is released from activated platelets upon their activation and regulates injury-induced angiogenesis, and Cyr61 which is critical for blood vessel formation in placenta and vascular integrity during embryonic development. The downregulation of HSPG2 (heparan sulfate proteoglycan 2), which binds extracellular heparin and regulates the vascular response to injury, is in accordance with former studies. We have previously characterized the role of coagulation pathways in the context of DXR-induced vascular toxicity, and the protective effect of enoxaparin, observed in DXR-treated mice [19]. Additionally, low molecular weight heparin was formerly found to reduce DXR-related renal toxicity [28]. This observation infer that the placental vascular toxicity is mediated by coagulation-related pathways and serve as a potential target for alleviating DXR-induced vascular toxicity. This could be appealing in the setting of cancer and pregnancy, due to the common use of these agents in both settings.

The immunohistochemical analysis of placental histological sections revealed higher rates of apoptosis and proliferation in the anthracycline-treated placentae and suggested that the treated placentae had overcompensated the damage, to reach the observed normal weights. To validate these data, we performed a preliminary retrospective clinical study. We examined placentae of seven patients, diagnosed with BC during pregnancy (weeks 27–35), who were treated with EPI, a 4’-epi-isomer of DXR, and matched them to ten chemo-naïve controls of the same gestational age. We found higher rates of apoptosis and proliferation as compared with in the mice placentae, and fewer newly formed blood vessels in the EPI-exposed human placentae. This difference in the neovascularization pattern between mice and human placentae could derive from differential recovery time from chemotherapy administrations to delivery.

Our study indicates that DXR and EPI exert an acute vascular impairment of the placental vasculature, manifested by reduced umbilical blood flow. Changes in the expression of biochemical markers in the placentae, depicted by immunohistochemistry and proteomic analysis six days after DXR administration, displayed pathways involved in endothelial barrier maintenance and coagulation. We suggest that the acute vascular impairment was preceded by a cascade of events that resulted in diminished placental function and smaller embryos. The long-term effect on the offspring should be further investigated.

Pregnant cancer patients who have been treated with chemotherapeutic drugs are at an increased risk of maternal venous thromboembolism (VTE) and placental vascular pathologies, which can progress into preeclampsia or fetal IUGR [12,13,14]. Revealing the mechanism of chemotherapy-induced placental toxicity could shed light on the role of vascular impairment. It could also result in revisiting current practice in order to apply protective measurements such as prophylactic anticoagulant, to reduce gestational complications. At present, there are no evidence-based recommendations for risk reduction of obstetrical and neonatal complications in pregnant patients diagnosed with cancer and treated with chemotherapy. Future studies should be planned to confirm the protective role of these agents.

## 4. Materials and Methods

### 4.1. Human Placentae Samples

The clinical protocol for the retrospective study was approved by the Fondazione IRCCS Ca’ Granda—Ospedale Maggiore Policlinico, Milan (protocol ID. S25470), which provided archival tissue blocks of placentae exposed to chemotherapy.

### 4.2. Animals

For in vivo animal studies, ICR (Institute of Cancer Research) female mice (Envigo RMS Limited, Jerusalem, Israel) were used. The eight-week-old female ICR mice were primed with 2.5 IU human chorionic gonadotropin (hCG) (Sigma, MO, USA) 48 h after administration of 2.5 IU pregnant mare serum gonadotropin (Syncro-part PMSG, Sanofi, Paris, France) and were allowed to mate. The following day was established as day E0.5 of pregnancy if a vaginal plug was detected. Ethical approval of animal experimentation was received from the Ethical Committee of the Sackler Faculty of Medicine, Tel-Aviv University (01-18-016).

### 4.3. Ultrasound Imaging

The mice were imaged on day E12.5 of pregnancy, which is equivalent to second trimester in women [29]. The molecular bioimaging platform enabled real-time evaluation of the same individual over time; hence, each mouse served as its own control. We followed our formerly published protocol [18]. Briefly, anesthetized mice were positioned on a MousePad, a 30 gauge, ½ inch needle attached to a 1 mL syringe was inserted into the tail vein for IV administration. An umbilical cord artery of one embryo was viewed by the PW Doppler mode of the high-resolution ultrasound (Vevo 2100, Visual Sonics, Toronto, Canada), with the transducer held immobilized, in position. Following a short stabilization period, a baseline arterial blood flow was recorded and quantified by analyzing the VTI, (mm) and heart rate interval (sec). Then, mice (~40 g BW) were injected IV (150 µL) with either DXR (8 mg/kg, *n* = 7, *n* = number of imaged arteries, one embryo per pregnant mouse) or saline (control, *n* = 6). The arterial blood flow was monitored continuously for 15 min, recorded and analyzed at various time points post injection. Analysis of each PW Doppler cine loop was performed, values of post-treatment imaging were normalized according to pretreatment imaging values of each mouse (defined as 100%). The saline-injected mice were standardized to 100% as a reference to DXR-injected mice. Only one embryo of one mother was checked (the one that was in a position that enabled stable monitoring); following the imaging, pregnant mice were returned to the animals’ facility.

### 4.4. Embryos and Placentae Weights

On day E18.5, which is equivalent to the end of the third trimester in women, 6 days after injection of either DXR or saline, the pregnant mice were euthanized and 10 minutes later embryos and placentae were removed from the uterus (*n* = 52, from 5 DXR-treated pregnant mice; *n* = 61, from 5 control pregnant mice). Each embryo and its placenta were weighed separately, before the placenta was dissected for further analysis.

### 4.5. Proteomics

Placentae from DXR- and saline-treated pregnant mice (*n* = 4, from 3 pregnant mice in each group) were dissected for proteomics analysis at the Smoler Proteomic Center, Technion, Israel. Proteolysis: tissues were homogenized with Omni-Th homogenizer (Omni International, Kennesaw, GA, USA) in urea buffer containing 8 M urea, 400 mM ammonium bicarbonate, and 10 mM DTT. Homogenates were sonicated (5’, (10/10 on/off pulses), 90% energy, Sonics Vibra-Cell, Sonics, Newtown, CT, USA) and briefly centrifuged to precipitate insoluble debris. The amount of protein was estimated using the Bradford assay. Twenty μg protein from each sample were reduced with DTT (60 °C for 30 min), modified with 40 mM iodoacetamide in 100 mM ammonium bicarbonate (in the dark, RT, 30’) and digested in 2 M urea, 100 mM ammonium bicarbonate with modified trypsin (Promega; Madison, WI, USA) overnight at 37 °C at a 1:50 enzyme-to-substrate ratio. A 4 h trypsin digestion was performed at 37 °C with a 1:100 enzyme-to-substrate ratio. Mass spectrometry (MS) analysis: the tryptic peptides were desalted using C18 tips (Harvard Apparatus, Holliston, MA, USA), dried and resuspended in 0.1% formic acid. The peptides were resolved by reverse-phase chromatography on 0.075 × 300 mm fused silica capillaries (J&W capillary columns, Agilent, Santa Clara, CA, USA) packed with Reprosil reversed phase material (Dr Maisch GmbH, Ammerbuch-Entringen , Germany). The peptides were eluted with linear 180 min gradient of 5% to 28%, 15 min gradient of 28% to 95%, and 25 min at 95% acetonitrile with 0.1% formic acid in water at a flow rate of 0.15 μL/min. MS was performed by Q Exactive plus mass spectrometer (Thermo Fisher Scientific, Waltham, MA, USA) in a positive mode, using repetitively full MS scan, followed by high collision dissociation (HCD) of the 10 most dominant ions selected from the first MS scan. The MS data from all the biological repeats were analyzed using the MaxQuant software 1.5.2.8 (Mathias Mann’s group, Munchan, Garmany) vs. the Mus-Musculus part of the Uniprot database, with 1% FDR. The data were quantified by label-free analysis using the same software, based on extracted ion currents (XICs) of peptides enabling quantitation from each LC/MS run for each peptide identified in any of the experiments. Statistical analysis: treatment effects were measured by the fold change between the averages of treatment group and control group. The effects were tested using a normal test on the log fold changes, with p-value adjustment to control the false discovery rate (FDR) across all tested proteins. Fold changes with FDR level of 0.05 or lower were considered significant. Physical and functional interactions between the statistically significant proteins were analyzed using STRING (Search Tool for Retrieval of Interacting Genes/Proteins) database [30].

### 4.6. Histology: Mouse and Human

We performed immunohistochemistry and TUNEL staining on placentae sections of mouse and human, as described previously [19]. In mice, placentae from pregnant females treated with either DXR (*n* = 52, from 4 pregnant mice) or saline (*n* = 21, from 4 pregnant mice); in humans, placentae from 7 women diagnosed with BC during pregnancy (week 27–35, Table S2) and treated with EPI, as well as human placentae from 10 chemo-naïve women (control), who were selected upon the delivery date to match with the study cohort. Moreover, we ruled out any existing vascular disorders in the control group (such as preeclampsia or known hypertension).

Briefly, placentae were excised and fixed in 4% paraformaldehyde, embedded in paraffin blocks and sectioned. Sections were deparaffinized, incubated over night at 4 °C with rabbit anti-proliferating cell nuclear antigen (PCNA 1:100; Santa Cruz Biotechnology, Santa Cruz, CA, USA), rat anti-cluster of differentiation (CD34 1:100; Cedarlane, Ontario, Canada), and mouse anti-pan-cytokeratin (1:100; Cell Marque, Roklin, CA, USA). We used Hoechst 33280 (1 µg/mL; Sigma Chemical) for DNA staining, mixed with the following secondary antibodies: Alexa-488-conjugated donkey anti-rabbit (1:200; Abcam, Cambridge, UK), Alexa-488-conjugated donkey anti-rat (1:200; Abcam), and Alexa-555-conjugated donkey anti-mouse (1:200; Abcam). DNA fragmentation was examined by TUNEL according to the manufacturer’s instructions (Dead End fluorometric TUNEL system; Promega). Positive control sections were exposed for 10 min to DNase I (6 units/mL; Invitrogen, Carlsbad, CA, USA). Bright-field images were recorded by a digital camera (Canon pc1089 CCD, Tokyo, Japan) connected to an Axiovert 200M inverted microscope (Carl Zeiss MicroImaging; Oberkochen, Germany) equipped with an Apochromat 20× objective. Florescence images were photographed by LSM-510 confocal laser-scanning microscope (CLSM; Carl Zeiss MicroImaging) equipped with a Plan-Neofluar 25× objective. Offset calibration of the photomultiplier was performed with sections stained with secondary antibodies only. Ki-67 staining of tonsil tissue served as positive control for immunoperoxidase staining. The average number of PCNA positive cells, TUNEL positive cells, or CD34 blood vessels were automatically analyzed by Fiji software (National Institutes of Health, Bethesda, MD, USA).

### 4.7. Data Analysis and Statistics

Data are expressed as mean ± SEM. Individual comparisons were made using a Student’s t-test. A *P* value of 0.05 was considered statistically significant. Professional statistical analysis was employed. Normal distribution was assessed by the Kolmogorov–Smirnov test.

## 5. Conclusions

Our present work unravels the damaging effect of anthracycline treatment during pregnancy; our findings indicate that placenta exposure to DXR or EPI compromises the blood supply to the embryo immediately after its administration, modifies its vascular-related pathways, thus, compromising its function and ultimately endangers the embryo.

## Supplementary Materials

The following are available online at https://www.mdpi.com/2072-6694/12/5/1277/s1, Table S1.: Embryos and Placentae Weights, Table S2.: Proteins exhibiting significant change in their expression upon exposure to DXR, Table S3.: Clinical Characteristics of Human Placentae Donors and Figure S1.: Histology of mice placentae.
