# Supplementary material for: Cancer During Pregnancy: The Role of Vascular Toxicity in Chemotherapy-Induced Placental Toxicity
Source: Cancers (Basel). 2020 May 18;12(5):1277. doi: 10.3390/cancers12051277 (PMC7281110; doi:10.3390/cancers12051277)
Supplement: Supplementary file 1 [file cancers-12-01277-s001.pdf]

Table S1. Embryos and Placentae Weights

Control weights (grams)					DXR weights (grams)				
		Embryo	Placenta	EPI			Embryo	Placenta	EPI
Mother 1	1	1.46	0.0864	16.8981	Mother 1	1	1.38	0.1165	11.85
	2	1.47	0.0964	15.2490		2	1.35	0.085	15.88
	3	1.49	0.1104	13.4964		3	1.28	0.1139	11.24
	4	1.54	0.1	15.4000		4	1.4	0.1097	12.76
	5	1.58	0.1038	15.2216		5	1.19	0.0916	12.99
	6	1.59	0.094	16.9149		6	1.26	0.0928	13.58
	7	1.34	0.0786	17.0483		7	1.29	0.1045	12.34
	8	1.28	0.0806	15.8809		8	1.31	0.0976	13.42
	9	1.47	0.0984	14.9390		9	1.18	0.0932	12.66
	10	1.51	0.097	15.5670		10	1.23	0.1125	10.93
	11	1.54	0.1031	14.9370		11	1.29	0.0721	17.89
	12	1.43	0.0955	14.9738		12	1.16	0.1085	10.69
	13	1.42	0.0993	14.3001					
					Mother 2	1	1.18	0.096	12.29
Mother 2	1	1.2581	0.0821	15.324		2	1.27	0.0838	15.16
	2	1.32	0.0936	14.1229		3	1.2	0.0962	12.47
	3	1.31	0.085	15.3647		4	1.16	0.0835	13.89
	4	1.36	0.0978	13.8855		5	1.18	0.0756	15.61
	5	1.22	0.0723	16.8783		6	1.19	0.0987	12.06
	6	1.33	0.0914	14.5810		7	1.23	0.0877	14.03
	7	1.33	0.1067	12.4555		8	1.3	0.0974	13.35
	8	1.34	0.089	15.0449		9	1.24	0.092	13.48
	9	1.22	0.0798	15.3321		10	1.28	0.08	16.00

	10	1.31	0.0974	13.4887
	11	1.36	0.0987	13.7923
	12	1.38	0.0742	18.6388
	13	1.28	0.1035	12.4145
	14	1.32	0.1082	12.2440
**Mother 3**	1	1.17	0.1228	9.5195
	2	1.36	0.1108	12.2292
	3	1.40	0.106	13.1849
	4	1.41	0.0915	15.4568
	5	1.32	0.1082	12.1738
	6	1.26	0.0967	13.0362
	7	1.30	0.1186	10.9941
	8	1.3667	0.1108	12.33484
**Mother 4**	1	1.09	0.0891	12.2492
	2	1.18	0.0955	12.4042
	3	1.06	0.081	13.0370
	4	1.18	0.1041	11.3112
	5	1.09	0.0918	11.8464
	6	1.24	0.0908	13.6696
	7	1.09	0.0893	12.1881
	8	1.08	0.0957	11.3271
	9	1.07	0.0965	11.1337
	10	1.14	0.0932	12.2146
	11	1.14	0.0902	12.6086

	11	1.21	0.0779	15.53
	12	1.17	0.1123	10.42
**Mother 3**	1	1.5803	0.1173	13.47
	2	1.2839	0.0968	13.26
	3	1.5382	0.108	14.24
	4	1.5047	0.1043	14.43
	5	1.524	0.1225	12.44
	6	1.4794	0.095	15.57
**Mother 4**	1	0.9003	0.081	11.11
	2	0.9938	0.1126	8.83
	3	0.8571	0.108	7.94
	4	0.7787	0.0718	10.85
	5	0.9911	0.0909	10.90
	6	1.1009	0.0881	12.50
	7	0.9081	0.0944	9.62
	8	0.8868	0.1054	8.41
	9	0.9781	0.1046	9.35
	10	1.0122	0.131	7.73
**Mother 5**	1	1.0154	0.0741	13.70
	2	1.024	0.0978	10.47
	3	0.967	0.0855	11.31
	4	0.0883	0.1011	0.87
	5	0.9976	0.0841	11.86

	12	1.15	0.0813	14.0898
	13	0.94	0.0714	13.1989
	14	1.16	0.099	11.7576
	15	1.19	0.0961	12.3725
	16	1.21	0.1212	9.9670
**Mother 5**	1	1.1576	0.0765	15.13203
	2	1.1544	0.0822	14.0438
	3	1.0743	0.096	11.19063
	4	1.0262	0.0882	11.63492
	5	1.1332	0.1006	11.26441
	6	1.1702	0.0893	13.10414
	7	1.1175	0.106	10.54245
	8	1.1937	0.0896	13.32254
	9	1.1937	0.0947	12.60507
	10	1.0739	0.1072	10.01772

	6	1.0278	0.0906	11.34
	7	0.852	0.0849	10.04
	8	0.9738	0.0742	13.12
	9	1.0779	0.0713	15.12

Table S2. Proteins exhibiting significant change in their expression upon exposure to DXR

Protein ID	Protein Names	Gene Name	Ratio of protein expression in DXR treated placentea vs. Control placentea	P Value
G5E893		Ankrd12	1.324771217	0
P35285;A2ARZ7	Ras-related protein Rab-22A	Rab22a	1.103485874	0
P28666	Murinoglobulin-2	Mug2	1.079858738	0
Q9EQI5		Ppbp	1.079301188	0
Q61646	Haptoglobin;Haptoglobin alpha chain;Haptoglobin beta chain	Hp	1.066084752	0
A0A1W2P8C6;P41731	CD63 antigen	Cd63	1.064040441	0
P18406	Protein CYR61	Cyr61	1.055193051	1.54E-12
Q80ZS9;Q08535;A0A1B0GS37	Secretin	Sct	1.046771605	4.54E-09
P52430;H3BK03;H3BLB8	Serum paraoxonase/arylesterase 1	Pon1	1.046478354	5.79E-09
E9Q6C2;Q8CG14	Complement C1s-A subcomponent;Complement C1s-A subcomponent heavy chain;Complement C1s-A subcomponent light chain	C1s1;C1sa	1.046150139	7.30E-09
Q62313;Q62314	Trans-Golgi network integral membrane protein 1;Trans-Golgi network integral membrane protein 2	Tgoln1;Tgoln2	1.04510771	1.81E-08
Q60590;P07361	Alpha-1-acid glycoprotein 1	Orm1	1.044140673	4.14E-08
A0A0A6YYE7;A0A0B4J1I9;A0A0B4J1I4;A0A0B4J1I3;A0A0A6YYE5;A0A075B5M4;A0A075B5L8		Igkv4-57-1;Igkv4-79	1.044012524	4.53E-08
Q921H9	Cytochrome c oxidase assembly factor 7	Coa7	1.043920998	4.81E-08

A0A0R4J182;Q9JL96;A0A286YE81;A0A286YCC4	Cathepsin M	Ctsm	1.043513648	6.70E-08
D3Z4L9;Q61333	Tumor necrosis factor alpha-induced protein 2	Tnfaip2	1.041257583	4.19E-07
E0CYQ4;E0CX64;H3BK26;P49442	Inositol polyphosphate 1-phosphatase	Inpp1	1.038369088	3.28E-06
Q91Y97	Fructose-bisphosphate aldolase B	Aldob	1.037879259	4.56E-06
A0A075B5P5;A0A1Y7VJN6;P03987	Ig gamma-3 chain C region	Ighg3	1.03783164	4.67E-06
Q3UDS7;A0A1L1SSF2;Q8VDL4	ADP-dependent glucokinase	Adpgk	1.03752025	5.82E-06
P56380	Bis(5-nucleosyl)-tetraphosphatase [asymmetrical]	Nudt2	1.037408954	6.16E-06
A0A075B5P3;A0A0A6YVP0		Ighg2b	1.037127328	7.40E-06
P13366;Q920S1	Granzyme G	Gzmg	1.036033943	1.53E-05
A0A0R4J0G0;Q8BH04;Q9Z2V4	Phosphoenolpyruvate carboxykinase [GTP], mitochondrial;Phosphoenolpyruvate carboxykinase, cytosolic [GTP]	Pck2;Pck1	1.034708415	3.82E-05
P21300	Aldose reductase-related protein 1	Akr1b7	1.034606934	4.05E-05
Q91VK1;A0A1Y7VM24;A0A1Y7VM47;A0A1Y7VIU9;A0A1Y7VKZ4;A0A1Y7VJ43	Basic leucine zipper and W2 domain-containing protein 2	Bzw2	1.034355438	4.69E-05
F8VQ42;E0CZ72;P28740	Kinesin-like protein KIF2A	Kif2a	1.034349308	4.69E-05
A0A0R4J1V1;Q75N73;D3Z6P5;D3Z354;D3YXD7	Zinc transporter ZIP14	Slc39a14	1.034109612	5.35E-05
Q80XD8	Proline-rich acidic protein 1	Prap1	1.033760948	6.53E-05
Q6P5D8	Structural maintenance of chromosomes flexible hinge domain-containing protein 1	Smchd1	1.033568202	7.29E-05
Q91XL1		Lrg1	1.033502974	7.47E-05
Q8WUR0;J3QPY0;A0A0U1RNT4	Protein C19orf12 homolog	1600014C10Rik	1.033291254	8.31E-05
A0A087WRL8;B8JK81;Q3TP05;B8JK87;Q8K371	Angiomotin-like protein 2	Amotl2	1.03206227	0.000182951
Q9CR95	Adaptin ear-binding coat-associated protein 1	Necap1	1.031886454	0.000203346
Q9Z0G0	PDZ domain-containing protein GIPC1	Gipc1	1.031777137	0.000214386

Q8R3H7	Heparan sulfate 2-O-sulfotransferase 1	Hs2st1	1.031323779	0.000277215
P70274;E0CZC5;E0CZ76;E0CX80	Selenoprotein P	Sepp1	1.031071883	0.000317481
O70238		Rhox6	1.030939557	0.000339585
Q9QZ25	Vascular non-inflammatory molecule 3	Vnn3	1.030512429	0.000430303
Q8BGS2	BolA-like protein 2	Bola2	1.030092444	0.000533251
Q8CIG8;A0A0R4J049;F6QQQ6	Protein arginine N-methyltransferase 5	Prmt5	1.029727139	0.000638401
Q9DBB9	Carboxypeptidase N subunit 2	Cpn2	1.029462851	0.000739422
Q8VDP3;E9PUI4	Protein-methionine sulfoxide oxidase MICAL1	Mical1	1.029407866	0.000759045
P08884	Granzyme E	Gzme	1.028857195	0.001007017
E9PUW7;Q9EPK7	Exportin-7	Xpo7	1.028602264	0.001148492
O89016;A0A1Y7VMF7	ATP-binding cassette sub-family D member 4	Abcd4	1.027986204	0.001617278
A0A1W2P766			1.027153391	0.002543768
E9Q3G8;A0A286YCP2		Nup153	1.026752483	0.00312882
F6XCT0		Macf1	1.026442435	0.003650235
E9Q5W5;A0A140LJ04;Q5SSH7	Zinc finger ZZ-type and EF-hand domain-containing protein 1	Zzef1	1.026139273	0.004210954
S4R2D5;A0A0A0MQN4;Q9D906;S4R2T5;S4R2P7;S4R1T0;S4R1K1;S4R1U3;S4R1W9;S4R1N8	Ubiquitin-like modifier-activating enzyme ATG7	Atg7	1.025959811	0.004618129
Q8BZA9;A0A0J9YV11	Fructose-2,6-bisphosphatase TIGAR	Tigar	1.025796999	0.004956989
P62046	Leucine-rich repeat and calponin homology domain-containing protein 1	Lrch1	1.025357888	0.006066764
Q3U816;Q9Z2G9	Oxidoreductase HTATIP2	Htatip2	1.025298291	0.006192569
Q9QXE7;A0A0J9YV90;Q8BHJ5	F-box-like/WD repeat-containing protein TBL1X;F-box-like/WD repeat-containing protein TBL1XR1	Tbl1x;Tbl1xr1	1.024972287	0.007282122
A2A7H1;Z4YN00;Q9QZN4;A2A7G9;A2A7H3	F-box only protein 6	Fbxo6	1.02492174	0.0073559
O70591;F8WJ30	Prefoldin subunit 2	Pfdn2	1.024867631	0.007446067
Q8BUU7;Q68FH4	N-acetylgalactosamine kinase	Galk2	1.024821782	0.007586956

F6RK81;F6UIS1;Q9CQE7	Endoplasmic reticulum-Golgi intermediate compartment protein 3	Ergic3	1.024739763	0.007814898
E9Q589;A0A0A6YY47;A0A0A6YY91;P13595;A0A0A6YWU2;E9QB01	Neural cell adhesion molecule 1	Ncam1	1.024463811	0.008736197
P02104	Hemoglobin subunit epsilon-Y2	Hbb-y	1.024461596	0.008736197
Q9D1C8	Vacuolar protein sorting-associated protein 28 homolog	Vps28	1.024337442	0.009249222
Q5PPR2;Q6P1Y9;Q8R3S6	Exocyst complex component 1	Exoc1	1.02411355	0.010094112
A0A075B5P4;A0A0A6YWR2;P01868;P01869	Ig gamma-1 chain C region secreted form;Ig gamma-1 chain C region, membrane-bound form	Ighg1	1.02397264	0.010795927
A0A0U1RPI8;Q9BCZ4;A0A0U1RP62	Selenoprotein S	Vimp	1.023857256	0.011338285
Q3TDD9	Protein phosphatase 1 regulatory subunit 21	Ppp1r21	1.023789984	0.011619209
P61294;A0A1L1SRS6	Ras-related protein Rab-6B	Rab6b	1.023528353	0.013197352
G3UZG8;G3UX07;G3UZH2;G3UY43;G3UX63	DNA (cytosine-5)-methyltransferase 3A	Dnmt3a	1.023441494	0.013719669
Q80YX1;Q80YX0	Tenascin	Tnc	1.023336749	0.014253321
Q64726	Zinc-alpha-2-glycoprotein	Azgp1	1.023197888	0.015061134
Q3TBA3;P21958;F6QHF1	Antigen peptide transporter 1	Tap1	1.023152943	0.015329742
Q3TCH7;E9PXT5;F6UV36	Cullin-4A	Cul4a	1.023095548	0.01561724
P28665	Murinoglobulin-1	Mug1	1.02294254	0.016402317
G3UZM9;Q8BWW9;G3UXH4;G3UYD6	Serine/threonine-protein kinase N2	Pkn2	1.022708829	0.017913915
Q3U0B3	Dehydrogenase/reductase SDR family member 11	Dhrs11	1.02249921	0.019280871
P16332	Methylmalonyl-CoA mutase, mitochondrial	Mut	1.022495967	0.019280871
Q9R0H0;A2A848	Peroxisomal acyl-coenzyme A oxidase 1	Acox1	1.022373286	0.020372717
Q3U5Q7	UMP-CMP kinase 2, mitochondrial	Cmpk2	1.021933582	0.024014269
O54831	Prolactin-7A2	Prl7a2	1.021900979	0.024114221
E9Q3M0;Q62432;E9Q5P9;E9PXA8;A2CG44;Q8BUN5;Q9JIW5;A2CG45;F7BUP2;Q8C3Y6;P97454;P70340	Mothers against decapentaplegic homolog 2;Mothers against decapentaplegic homolog 3;Mothers against decapentaplegic homolog 9	Smad2;Smad3;Smad9	1.021807198	0.025009363

Q19LI2	Alpha-1B-glycoprotein	A1bg	1.021693655	0.026280084
Q8BVU5;A2AH28;A2AH27	ADP-ribose pyrophosphatase, mitochondrial	Nudt9	1.021534843	0.028196265
B2RSU6;Q6AW69;D3Z3H4	Cingulin-like protein 1	Cgnl1	1.021507425	0.028220274
Q9R112;F6ZKZ3;H3BLH2	Sulfide:quinone oxidoreductase, mitochondrial	Sqrdl	1.0213283	0.030104788
E9Q6J4		Ceacam3	1.021244144	0.030948791
P62309	Small nuclear ribonucleoprotein G	Snrpg	1.021137689	0.032264266
P09813	Apolipoprotein A-II;Proapolipoprotein A-II	Apoa2	1.021063994	0.033251956
E9QNR6;Q8VD62	UPF0696 protein C11orf68 homolog	s03	1.020823755	0.036971208
Q3UGS4	Protein FAM195B	Fam195b	1.020750419	0.038082138
Q91XF0;A2A6E7	Pyridoxine-5-phosphate oxidase	Pnpo	1.020654018	0.039634065
Q9Z247	Peptidyl-prolyl cis-trans isomerase FKBP9	Fkbp9	1.020594439	0.040564963
A2AQ07;CON__ENSEMBL:ENSBTAP00000025008	Tubulin beta-1 chain	Tubb1	1.020291258	0.045145501
E9Q8N1;E9Q8K5;A2ASS6;A2AT70;F7CR78	Titin	Ttn	1.020204658	0.046586109
P46737;A3KGA8	Lys-63-specific deubiquitinase BRCC36	Brcc3	1.020161676	0.047149984
P04444;P04443	Hemoglobin subunit beta-H1	Hbb-bh1	1.020139196	0.047455531
A0A0G2JER6;F8VQE9;Q8VHH5	Arf-GAP with GTPase, ANK repeat and PH domain-containing protein 3	Agap3	1.020096756	0.048186278
Q60994;E9PWU4	Adiponectin	Adipoq	1.020037156	0.049298722
D3Z0T5;Q8BQ47	Protein canopy homolog 4	Cnpy4	0.980164283	0.04690464
E9Q1D5;Q62219	Transforming growth factor beta-1-induced transcript 1 protein	Tgfb1i1	0.980115326	0.046180308
Q9Z2D6	Methyl-CpG-binding protein 2	Mecp2	0.979985468	0.043809052
Q9DBC3;G3UZ59;E0CXC2	Cap-specific mRNA (nucleoside-2-O-)-methyltransferase 1	Cmtr1	0.979953862	0.043462192
P97742	Carnitine O-palmitoyltransferase 1, liver isoform	Cpt1a	0.979922252	0.042980986
A0A087WRH2;Q9WUD8;D3Z3C1	Fas apoptotic inhibitory molecule 1	Faim	0.979896529	0.042621724
Q91VH6	Protein MEMO1	Memo1	0.979892045	0.042621724

A0A0U1RP47;A0A0U1RQ85;P22682;A0A0X1KG61	E3 ubiquitin-protein ligase CBL	Cbl	0.979794377	0.040928826
Q9QUR7;Q3ULQ2	Peptidyl-prolyl cis-trans isomerase NIMA-interacting 1	Pin1;Pin1rt1	0.979226292	0.032074655
A2AWA9;A2AWA7;A2AWB0	Rab GTPase-activating protein 1	Rabgap1	0.97914004	0.030948791
Q9DBR0	A-kinase anchor protein 8	Akap8	0.97913283	0.030948791
Q99NB1	Acetyl-coenzyme A synthetase 2-like, mitochondrial	Acss1	0.978986155	0.029107083
Q8VHL1	Histone-lysine N-methyltransferase SETD7	Setd7	0.978925168	0.028365239
P11031	Activated RNA polymerase II transcriptional coactivator p15	Sub1	0.978914949	0.028332698
P32883	GTPase KRas;GTPase KRas, N-terminally processed	Kras	0.978898895	0.028220274
Q9CQ22;A0A0A6YX02	Ragulator complex protein LAMTOR1	Lamtor1	0.978893956	0.028220274
Q9Z1T2;A0A1D5RMB8;CON__ENSEMBL:ENSBTAP00000006074;Q9R0G6	Thrombospondin-4;Cartilage oligomeric matrix protein	Thbs4;Comp	0.978873327	0.028196265
P32233	Developmentally-regulated GTP-binding protein 1	Drg1	0.978579693	0.024800368
E9PWQ3;J3QQ16;A0A087WS16;D3YWD1		Col6a3	0.978498473	0.024014269
Q6NZR5;G3UYT1		Skiv2l	0.978490985	0.024014269
Q9CQN1	Heat shock protein 75 kDa, mitochondrial	Trap1	0.97841144	0.023277853
Q920E5;A0A0G2JEA5;A0A0G2JDJ5;A0A0G2JEB3;A0A0G2JE82	Farnesyl pyrophosphate synthase	Fdps	0.978390436	0.023129317
O08759;A0A0J9YUY4;A0A0J9YVG1;A0A0J9YUK0	Ubiquitin-protein ligase E3A	Ube3a	0.978367203	0.022956412
Q9QYE6;A0A1Y7VMD3	Golgin subfamily A member 5	Golga5	0.978309663	0.022398465
P41233;E9Q6G4;Q91V24	ATP-binding cassette sub-family A member 1	Abca1	0.978183692	0.021203509
D3Z7S0;D3Z4J5;Q9D1H7	Golgi to ER traffic protein 4 homolog	Get4	0.978156801	0.02109289
Q9CZR8;Q9CX33	Elongation factor Ts, mitochondrial	Tsfm	0.978129938	0.020896835
A2AAN0;O35250;A2AAN3	Exocyst complex component 7	Exoc7	0.978109247	0.020767044
Q9DAR7;Q3TBW9;D6RFQ0	m7GpppX diphosphatase	Dcps	0.977923082	0.019225315
Q80V42	Carboxypeptidase M	Cpm	0.977910337	0.019183096

CON__P04264			0.977884994	0.019019253
F8WH23;E9Q5D9;Q61581	Insulin-like growth factor-binding protein 7	Igfbp7	0.977881668	0.019019253
A0A1Y7VKN7;A0A1Y7VL28;Q80XC2	tRNA (adenine(58)-N(1))-methyltransferase catalytic subunit TRMT61A	Trmt61a	0.977782237	0.018206745
E9Q6Q8;E0CX53;Q8BYJ6;H7BX82;F6SUV7	TBC1 domain family member 4	Tbc1d4	0.977730393	0.017886172
P97470;A0A0U1RPR5	Serine/threonine-protein phosphatase 4 catalytic subunit	Ppp4c	0.977712727	0.017803153
Q922F4	Tubulin beta-6 chain	Tubb6	0.977575739	0.016668829
P58742	Aladin	Aaas	0.977568604	0.016668829
Q80VP1	Epsin-1	Epn1	0.977548308	0.016581379
Q6PAV2	Probable E3 ubiquitin-protein ligase HERC4	Herc4	0.977461004	0.015993763
Q3U0I9;Q8CFD4;A0A0G2JDJ6;A0A0G2JEQ9	Sorting nexin-8	Snx8	0.977441271	0.015902846
P82198;Q3UXJ2	Transforming growth factor-beta-induced protein ig-h3	Tgfbi	0.977401114	0.015646397
Q4PZA2	Endothelin-converting enzyme 1	Ece1	0.977388757	0.015617265
Q923T9;A0A286YCW8;A0A286YCH4	Calcium/calmodulin-dependent protein kinase type II subunit gamma	Camk2g	0.97737996	0.01561724
Q5JC28;H3BK65;P42567;F6W2Q5;A0A0R4J0A0;H3BJB8	Epidermal growth factor receptor substrate 15	Eps15	0.977326503	0.015330174
Q920I9	WD repeat-containing protein 7	Wdr7	0.977256144	0.014986662
Q9D566;P49891	Sulfotransferase;Estrogen sulfotransferase, testis isoform	Sult1e1	0.977192732	0.014567385
Q6PGL7;A0A0N4SUJ0;A0A0N4SV74	WASH complex subunit FAM21	Fam21	0.977113653	0.014109886
Q8BMT0		Serpinb9d	0.977103112	0.014099018
Q00519;G3X982;Q3TYQ9	Xanthine dehydrogenase/oxidase;Xanthine dehydrogenase;Xanthine oxidase	Xdh	0.976660626	0.011376085
O08738	Caspase-6;Caspase-6 subunit p18;Caspase-6 subunit p11	Casp6	0.976611161	0.011188415
Q02788;D3Z7D5	Collagen alpha-2(VI) chain	Col6a2	0.976350071	0.009870522
A2AFQ0;Q7TMY8;F6XP90;F6UYC1	E3 ubiquitin-protein ligase HUWE1	Huwe1	0.976311555	0.009715737
Q9D7H3;D6RD00;D6REI7;D3Z263	RNA 3-terminal phosphate cyclase	RtcA;Rtca	0.976273196	0.009564129
A0A0R4J0I1			0.976270572	0.009564129

Q9CWQ8;Q8CAB8	GATS-like protein 3	Gatsl3	0.976007641	0.008535361
Q9D1M7	Peptidyl-prolyl cis-trans isomerase FKBP11	Fkbp11	0.976004548	0.008535361
Q9CQW5	Galectin-2	Lgals2	0.975919716	0.008219379
Q9CQ10;A0A0N4SVS3;A0A0N4SUX9	Charged multivesicular body protein 3	Chmp3	0.975852667	0.007963952
A2AA71;Q3U2P1;A2AA72	Protein transport protein Sec24A	Sec24a	0.975808743	0.007814898
P50285;Q3UNX7;D3Z0T2;A0A0A6YVP9;Q8C9C1	Dimethylaniline monooxygenase [N-oxide-forming] 1	Fmo1	0.975776686	0.007760171
Q99JY8	Lipid phosphate phosphohydrolase 3	Ppap2b	0.97567569	0.007446067
D5MCW4;Q9CQ89	Protein CutA	Cuta	0.975656884	0.007421762
Q8BWM0	Prostaglandin E synthase 2;Prostaglandin E synthase 2 truncated form	Ptges2	0.975620782	0.007355105
Q91WK1	SPRY domain-containing protein 4	Spryd4	0.9755954	0.007292521
Q8BH69	Selenide, water dikinase 1	Sephs1	0.975463772	0.006853517
A6H5Z3	Exocyst complex component 6B	Exoc6b	0.975194282	0.006027715
Q6S9I0;Q6S9I3		Kng2	0.975093248	0.005726376
Q9Z160;Q810S7;F6QQB5;A2A6N9;F6RK91;F6V3F1	Conserved oligomeric Golgi complex subunit 1	Cog1	0.975060341	0.005653378
Q91W97	Putative hexokinase HKDC1	Hkdc1	0.975058423	0.005653378
Q91W96;D6RDT6	Anaphase-promoting complex subunit 4	Anapc4	0.975028965	0.005619076
O88986;E9PWY6	2-amino-3-ketobutyrate coenzyme A ligase, mitochondrial	Gcat	0.974752304	0.004856491
A0A087WRE5;Q6P3D0;A0A087WSB5;E9Q9G1;Q8VHN8	U8 snoRNA-decapping enzyme	Nudt16	0.974381808	0.004017765
D3YXQ6		Psg16	0.97428743	0.003826487
Q99MR3	Solute carrier family 12 member 9	Slc12a9	0.974239815	0.003744672
Q3V493;Q9ES56	Trafficking protein particle complex subunit 4	Trappc4	0.97403545	0.00336369
Q9JKK1;A0A0A6YXG0;A0A0A6YW23	Syntaxin-6	Stx6	0.974021313	0.00335682
Q80SX7;Q8BXL7;V9GX20;E9PZK7	ADP-ribosylation factor-related protein 1	Arfrp1	0.973632262	0.002699837

A0A0R3P9C8;Q9DC69	NADH dehydrogenase [ubiquinone] 1 alpha subcomplex subunit 9, mitochondrial	Ndufa9	0.973581479	0.002636
E9QAU4;A2AQ25;D3Z781;Q8BHR1;B1AY50;B1AY49	Sickle tail protein	Etl4;Skt	0.973402403	0.002398441
Q9JK53	Prolargin	Prelp	0.973255859	0.002209977
Q8C142	Low density lipoprotein receptor adapter protein 1	Ldlrap1	0.972786772	0.001671403
Q3UIL6;A0A1L1SQ79;S4R243	Pleckstrin homology domain-containing family A member 7	Plekha7	0.972447556	0.001373369
Q8VCM3;A0A1Y7VLE1;A0A1Y7VNH4;A0A1Y7VJT0	Zinc finger FYVE domain-containing protein 21	Zfyve21	0.972424664	0.001363042
Q9Z2A7;F6V8W6	Diacylglycerol O-acyltransferase 1	Dgat1	0.971944437	0.001022259
Q3TCU5;Q9R233;G3UZZ2	Tapasin	Tapbp	0.971932947	0.001021809
Q9JJE7	Fatty acid desaturase 3	Fads3	0.971744842	0.000919424
Q63810	Calcineurin subunit B type 1	Ppp3r1	0.971687403	0.000892577
E9QQ99;D3Z1Y1;A0A0R4J1R1;Q9DBY1;D3YZH4	E3 ubiquitin-protein ligase synoviolin	Syvn1	0.971593136	0.000846327
Q3TAW3;E9Q296;Q8C178;Q9EPL9;A0A0J9YVH1;D3Z2N6	Acyl-coenzyme A oxidase;Peroxisomal acyl-coenzyme A oxidase 3	Acox3	0.971572073	0.000840915
P70227;P11881	Inositol 1,4,5-trisphosphate receptor type 3	Itpr3	0.97142875	0.000772486
Q9D6A7;I7HJI5;J3JSA2		Serpinb9c	0.971102579	0.000643939
Q6P2B1;F6TDZ2;E9Q0H8;A0A0U1RQ41;Q8K368	Transportin-3	Tnpo3	0.971021306	0.000619857
Q80UG2;P70207	Plexin-A4	Plxna4	0.971012757	0.000619857
D3Z041;P41216;D3Z457;D3YVF6;F6WNZ2	Long-chain-fatty-acid--CoA ligase 1	Acsl1	0.970982575	0.000613544
Q3TCD4;Q9WUR2;E9Q7A8;E9PYC6;E9Q858;E9PUY9;E9PYA9;E9PVM6;E9PY42;Q78JN3	Enoyl-CoA delta isomerase 2, mitochondrial	Eci2	0.970825502	0.000557815
A0A0G2JGD2;P07091	Protein S100-A4	S100a4	0.970791922	0.000549846
Q8C147	Dedicator of cytokinesis protein 8	Dock8	0.970684012	0.000520148
Q9DBG7;F6VF36	Signal recognition particle receptor subunit alpha	Srpr	0.9706782	0.000520148

Q06138;Q9DB16	Calcium-binding protein 39	Cab39	0.970625495	0.000508385
P70663	SPARC-like protein 1	Sparcl1	0.97057715	0.000496331
Q811S7	Upstream-binding protein 1	Ubp1	0.97038049	0.000439102
F8WGI9;Q91YY0;P98156;F8WIN7	Very low-density lipoprotein receptor	Vldlr	0.970323525	0.000429672
Q5SRX1;Q5SXA5;Q5SXA4	TOM1-like protein 2	Tom1l2	0.970219408	0.000404105
O35114	Lysosome membrane protein 2	Scarb2	0.970035607	0.000360169
Q8R5A6;Q80VE5	TBC1 domain family member 22A	Tbc1d22a;Tbc1d22b	0.969894384	0.000332976
Q61176	Arginase-1	Arg1	0.969586868	0.000277215
Q9QZ82;A0A087WRU6	Cholesterol side-chain cleavage enzyme, mitochondrial	Cyp11a1	0.969483718	0.000262898
Q91XC9	Peroxisomal membrane protein PEX16	Pex16	0.969293536	0.000232742
A2AJY7;A2AJY5;A2AJY2;O35206	Collagen alpha-1(XV) chain;Restin	Col15a1	0.969177414	0.00021674
Q62384;F8WHU9	Zinc finger protein ZPR1	Zpr1	0.969143882	0.000214386
Q04207;A4QPD3;P15307	Transcription factor p65;Proto-oncogene c-Rel	Rela;Rel	0.968349296	0.00012702
Q9DAU1;B0V2V1	Protein canopy homolog 3	Cnpy3	0.968263579	0.000120649
Q9EQ09;E0CXW6	Oxidized low-density lipoprotein receptor 1;Oxidized low-density lipoprotein receptor 1, soluble form	Olr1	0.967731348	8.31E-05
E9Q5B2;G5E8X1;Q9DCS2;D3YZM4;E9Q7K5	UPF0585 protein C16orf13 homolog	0610011F06Rik	0.967714621	8.31E-05
F6Z4F8;A2A7F7;Q9WU20;A2A7F9	Methylenetetrahydrofolate reductase	Mthfr	0.967686084	8.29E-05
Q8JZV7;F6UP77;F7CUP3	Putative N-acetylglucosamine-6-phosphate deacetylase	Amdhd2	0.967500006	7.38E-05
A0A087WS27;D3Z5S8		Fam46a	0.967334206	6.68E-05
D3Z383;Q07646	Mesoderm-specific transcript protein	Mest	0.967279923	6.53E-05
Q8BW75;E9Q3X6;E9PVL9	Amine oxidase [flavin-containing] B	Maob	0.967251587	6.48E-05
A0A0G2JFM9;E9PUK3;E9PUI5;Q8K012	Formin-binding protein 1-like	Fnbp1l	0.967069737	5.73E-05
F7C386;F6TVX7;Q8BSF4;E9PX91;D3YY63	Phosphatidylserine decarboxylase proenzyme;Phosphatidylserine decarboxylase alpha chain;Phosphatidylserine decarboxylase beta chain	Pisd;Gm20671	0.966837483	4.93E-05

O88531;B1B0P8;B1B0P9	Palmitoyl-protein thioesterase 1	Ppt1	0.966537348	4.13E-05
O35551;Q3U983;J3QJV7	Rab GTPase-binding effector protein 1	Rabep1	0.966261188	3.47E-05
A2A5N7;D3Z6V6;Q8BTJ4	Bis(5-adenosyl)-triphosphatase enpp4	Enpp7;Enpp4	0.965998587	2.88E-05
Q64449;A2AAA9	C-type mannose receptor 2	Mrc2	0.965090245	1.46E-05
Q9WUR9;A2ARF6;F6TEU8	Adenylate kinase 4, mitochondrial	Ak4	0.965017586	1.40E-05
Q3U125;Q9CYH2;D3Z0A8;D3YYG8;D3Z398;D3Z252;D3Z143;D3YWL4	Redox-regulatory protein FAM213A	Fam213a	0.965001338	1.40E-05
A0A0R4J1G5;Q91X78	Erlin-1	Erlin1	0.9646569	1.08E-05
Q99LT0	Protein dpy-30 homolog	Dpy30	0.964557032	1.01E-05
Q5SUF2;F8WGA8	Luc7-like protein 3	Luc7l3	0.963981493	6.56E-06
F8WHG5;Q60823	RAC-beta serine/threonine-protein kinase	Akt2	0.96384603	6.04E-06
A0A1L1SRW7;A0A1L1SS90;A0A0R4J050;Q99JW2;A0A1L1SRC1;A0A1L1SRL5;A0A087WPX1;A0A087WRE0;A0A1L1SSU4	Aminoacylase-1	Acy1	0.963375697	4.35E-06
F8WJI3;Q8R3Q6;E9Q4B5	Coiled-coil domain-containing protein 58	Ccdc58	0.963040439	3.35E-06
Q3V0K9;D3Z6J7	Plastin-1	Pls1	0.962889946	3.05E-06
Q8VCR7;A0A087WPF8;A0A087WSR2;E9QN99;A0A087WP24;A0A087WRJ2;A0A1L1SRH5;A0A1L1SUX1	Alpha/beta hydrolase domain-containing protein 14B	Abhd14b	0.9625471	2.33E-06
Q9DAZ2;Q5SXK2	Prolactin-2B1	Prl2b1	0.961754624	1.21E-06
A0A0X1KG62;Q8C4Y3;F6UFF7;F6SWS9	Negative elongation factor B	Nelfb	0.961522814	1.01E-06
E9QKZ2;Q91YE6;E0CXB2;F6TLX3;E0CY46;F6UT58	Importin-9	Ipo9	0.961365749	8.92E-07
J3QNK8;Q8CBW3;A0A087WP64;A0A087WPE6;A0A087WNT3;A0A087WNU9;A0A087WRS3;A0A087WPP8;Q6AXH6;Q6AXD2;P62484	Abl interactor 1	Abi1	0.961324888	8.75E-07
Q9ER81	Torsin-1A-interacting protein 2, isoform IFRG15	Tor1aip2	0.961281079	8.57E-07
P47857	ATP-dependent 6-phosphofructokinase, muscle type	Pfkm	0.961176101	7.95E-07

Q6PB93	Polypeptide N-acetylgalactosaminyltransferase 2;Polypeptide N-acetylgalactosaminyltransferase 2 soluble form	Galnt2	0.961165845	7.95E-07
F6Q3K8;Q924M7	Mannose-6-phosphate isomerase	Mpi	0.960830666	6.09E-07
Q62056		Psg17	0.960792703	6.00E-07
Q9Z110;D3Z0B4	Delta-1-pyrroline-5-carboxylate synthase;Glutamate 5-kinase;Gamma-glutamyl phosphate reductase	Aldh18a1	0.960701473	5.64E-07
Q3U0V2	Tumor necrosis factor receptor type 1-associated DEATH domain protein	Tradd	0.960515491	4.87E-07
Q61805;A2AC65;F6XKX9	Lipopolysaccharide-binding protein	Lbp	0.959575845	2.18E-07
Q80WQ2;A0A1D5RLY2	Protein VAC14 homolog	Vac14	0.959094301	1.44E-07
Q9JHW9;G3UWP3;A0A140LIF9;Q62148	Aldehyde dehydrogenase family 1 member A3	Aldh1a3	0.955836375	7.30E-09
P51432	1-phosphatidylinositol 4,5-bisphosphate phosphodiesterase beta-3	Plcb3	0.955614867	6.13E-09
Q3UTR7;P11859	Angiotensinogen;Angiotensin-1;Angiotensin-2;Angiotensin-3;Angiotensin-4;Angiotensin 1-9;Angiotensin 1-7;Angiotensin 1-5;Angiotensin 1-4	Agt	0.954807666	2.93E-09
Q9DBN5;Q8BK80	Lon protease homolog 2, peroxisomal	Lonp2	0.954211565	1.63E-09
A0A075B6A3;A0A0A6YXW6		Igha	0.953890214	1.20E-09
B7ZC23;B7ZC24;Q91W39	Nuclear receptor coactivator 5	Ncoa5	0.951375849	8.74E-11
Q6P8N8;Q9JHS4;F7BB92	ATP-dependent Clp protease ATP-binding subunit clpX-like, mitochondrial	Clpx	0.950775693	4.64E-11
D3Z6W7;Q3UHH8	Glucoside xylosyltransferase 1	Gxylt1	0.949341752	9.54E-12
A0A0R4J0P1;Q9D7B6;D3YTT4;A0A1L1SUG2	Isobutyryl-CoA dehydrogenase, mitochondrial	Acad8	0.949151707	7.91E-12
H3BJ30;H3BJW3;Q6NVF9;H3BKW0	Cleavage and polyadenylation specificity factor subunit 6	Cpsf6	0.948418032	3.49E-12
E9Q6L4;Q60949	TBC1 domain family member 1	Tbc1d1	0.948381575	3.46E-12
Q61823	Programmed cell death protein 4	Pdcd4	0.947810966	1.82E-12
E9PZ88;F8WIE1;Q91W89;E9PYM7;F6TFL1	Alpha-mannosidase 2C1	Man2c1	0.946474484	4.06E-13
Q7M732	Retrotransposon-like protein 1	Rtl1	0.946404437	3.69E-13

A0A087WR20;P49935	Pro-cathepsin H;Cathepsin H mini chain;Cathepsin H;Cathepsin H heavy chain;Cathepsin H light chain	Ctsh	0.943031552	0
Q6PKB0;Q61559;A0A1B0GSK0	IgG receptor FcRn large subunit p51	Fcgrt	0.941745827	0
Q9JLN9	Serine/threonine-protein kinase mTOR	Mtor	0.939012815	0
Q9Z315	U4/U6.U5 tri-snRNP-associated protein 1	Sart1	0.934059022	0
CON__P13645;A2A513;CON__P02535-1;P02535;CON__Q148H6;CON__Q7Z3Y7;CON__Q7Z3Z0;CON__Q7Z3Y8			0.930688563	0
Q3U487	E3 ubiquitin-protein ligase HECTD3	Hectd3	0.927746563	0
A2ARV4;A2ARV5	Low-density lipoprotein receptor-related protein 2	Lrp2	0.924717577	0
Q9D3P8;D3YXU2;D3YY76;D3YV19	Plasminogen receptor (KT)	Plgrkt	0.92373798	0
O54950;D3YUS1;Q8BIQ9;Q91WG5	5-AMP-activated protein kinase subunit gamma-1	Prkag1	0.920449787	0
O08601	Microsomal triglyceride transfer protein large subunit	Mttp	0.920334783	0
P70695	Fructose-1,6-bisphosphatase isozyme 2	Fbp2	0.917260255	0
CON__P48668;CON__P04259;CON__P02538;CON__P19013			0.910125464	0
Q9JLB4	Cubilin	Cubn	0.908571976	0
Q9CQ40	39S ribosomal protein L49, mitochondrial	Mrpl49	0.905094094	0
Q99JB7	Protein amnionless	Amn	0.900314347	0
Q60960;G3UXF0	Importin subunit alpha-5;Importin subunit alpha-5, N-terminally processed	Kpna1	0.890951568	0
Q05793	Basement membrane-specific heparan sulfate proteoglycan core protein;Endorepellin;LG3 peptide	Hspg2	0.860502184	0
O08712	Tumor necrosis factor receptor superfamily member 11B	Tnfrsf11b	0.852761721	0

Table S3. Clinical Characteristics of Human Placentae Donors

Maternal age at delivery (yrs)	Tumor stage (AGCC 2107)	Surgery during pregnancy	Chemo during pregnancy	Epirubicin total dose (mg/sqm)	Sex and kindred	Gestational age at delivery (wks)	Baby birth weight (g)
33	IIA	Biopsy	EC x 6	450	M G1	40	2690 (SGA)
37	IIA	Quad + axilla	EC x 4	300	F G3	37	2660 (AGA)
38	IIB	Mast + axilla	EC x 2	150	M G1	36	3635 (AGA)
32	IIB	Biopsy	EC x 3	225	M G2	39	3180 (AGA)
35	IIA	Biopsy	EC x 4	300	F G2	38	3070 (AGA)
41	IA	Quad + axilla	wE x 8	280	M G1	38	2825 (AGA)
37	IIIA	Mast + axilla	EC x 3	225	F G2	36	2830 (AGA)

Abbreviations:

EC: Epirubicin/Cyclophosphamide; wE: weekly Epirubicin; M: male; F: female; G: number of kindred;

SGA: small for gestational age*; AGA: appropriate for gestational age*

*according to Bertino E, Spada E, Occhi L et al. Neonatal anthropometric charts: the Italian neonatal study. J Pediatr Gastroenterol Nutr 2010;51 (3):353-61.

Figure S1. Histology of mice placentae

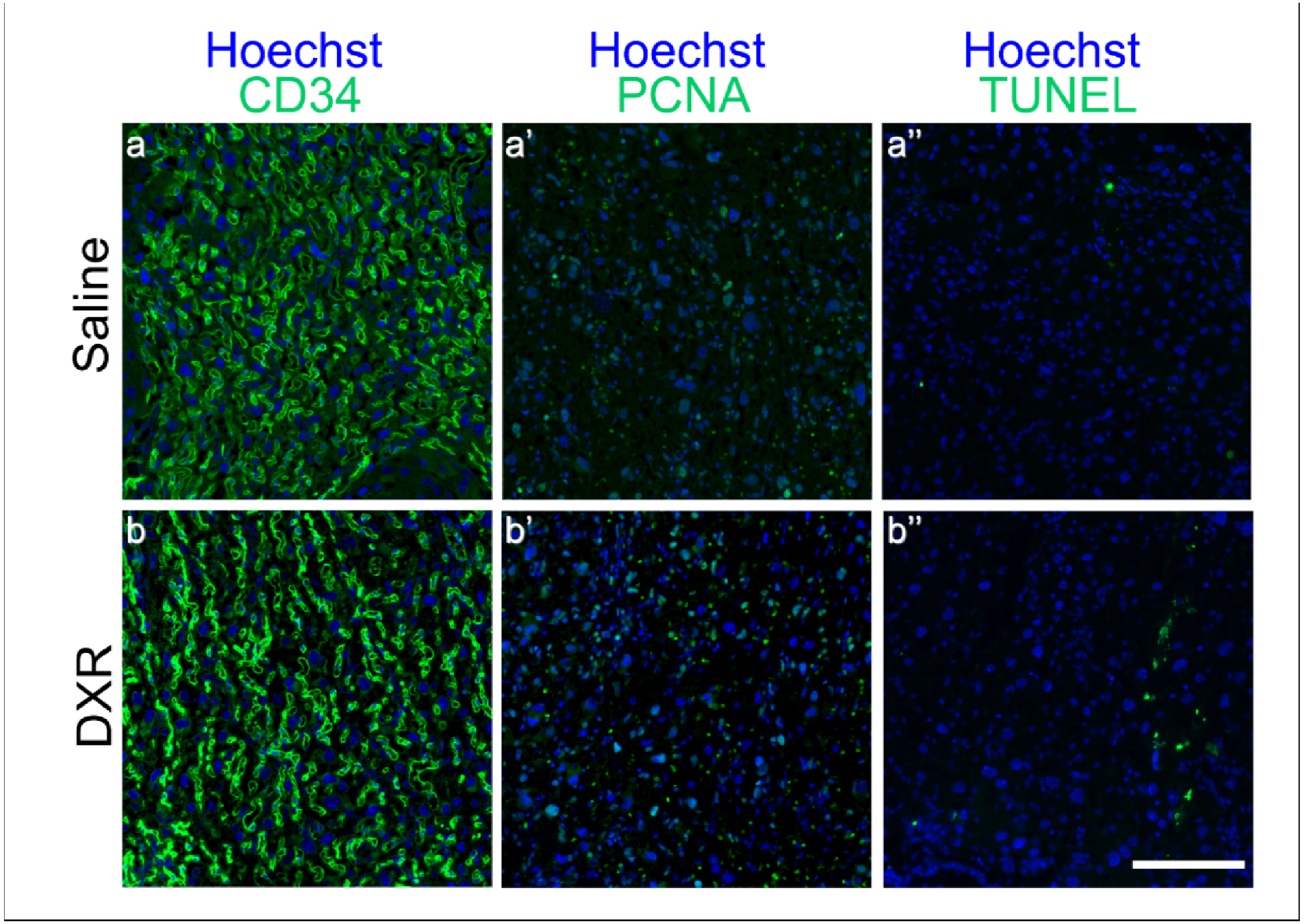

Representative pictures of control- (a-a'') or DXR-exposed (b-b'') mice-placenta, stained for CD34 (neovascularization; a,b), PCNA (proliferation; a',b') or TUNEL (apoptosis; a''',b'''). Florescence images were photographed by LSM-510 confocal laser-scanning microscope; offset calibration of the hotomultiplier was performed with sections stained with secondary antibodies only. Bar = 100 µm.
